# Managing hyperemesis gravidarum: a multimodal challenge

**DOI:** 10.1186/1741-7015-8-46

**Published:** 2010-07-15

**Authors:** JK Jueckstock, R Kaestner, I Mylonas

**Affiliations:** 1 First Department of Obstetrics and Gynaecology, Campus Innenstadt, Ludwig-Maximilians-University, Maistrasse 11, 80337 Munich, Germany

## Abstract

Up to 90% of pregnant women experience nausea and vomiting. When prolonged or severe, this is known as hyperemesis gravidarum (HG), which can, in individual cases, be life threatening. In this article the aetiology, diagnosis and treatment strategies will be presented based on a selective literature review. Treatment strategies range from outpatient dietary advice and antiemetic drugs to hospitalization and intravenous (IV) fluid replacement in persistent or severe cases. Alternative methods, such as acupuncture, are not yet evidence based but sometimes have a therapeutic effect.

In most cases, the condition is self limiting and subsides by around 20 weeks gestation. More severe forms require medical intervention once other organic causes of nausea and vomiting have been excluded. In addition, a psychosomatic approach is often helpful.

In view of its potential complexity, general practitioners and obstetricians should be well informed about HG and therapy should be multimodal.

## Introduction

About 50% - 90% of all pregnancies are accompanied by nausea and vomiting [1]. According to a study of more than 360 pregnant women, only 2% experienced only nausea in the morning whereas, in 80%, complaints persisted throughout the day. The condition is usually self-limiting and peaks at around 9 weeks gestation. At 20 weeks symptoms typically cease. However, in up to 20% of cases, nausea and vomiting may continue until delivery [1].

This condition is known as nausea and vomiting during pregnancy (NVP) or emesis gravidarum and is of no pathological significance as long as the affected women do not feel unwell or restricted in their daily life [2]. There are, however, different grades in the scope of NVP, which range from occasional morning-sickness to excessive vomiting that persists throughout the day. The most severe grade of NVP often leads to hyperemesis gravidarum (HG; see below), but it can be difficult to differentiate between the two conditions.

A prospective study of more than 9000 pregnant women showed that NVP occurred significantly more often in primigravidas and in women who were less educated, younger, non-smokers and overweight or obese. The incidence of NVP was also higher in women with a history of nausea and vomiting in a previous pregnancy [3].

**Figure 1:**
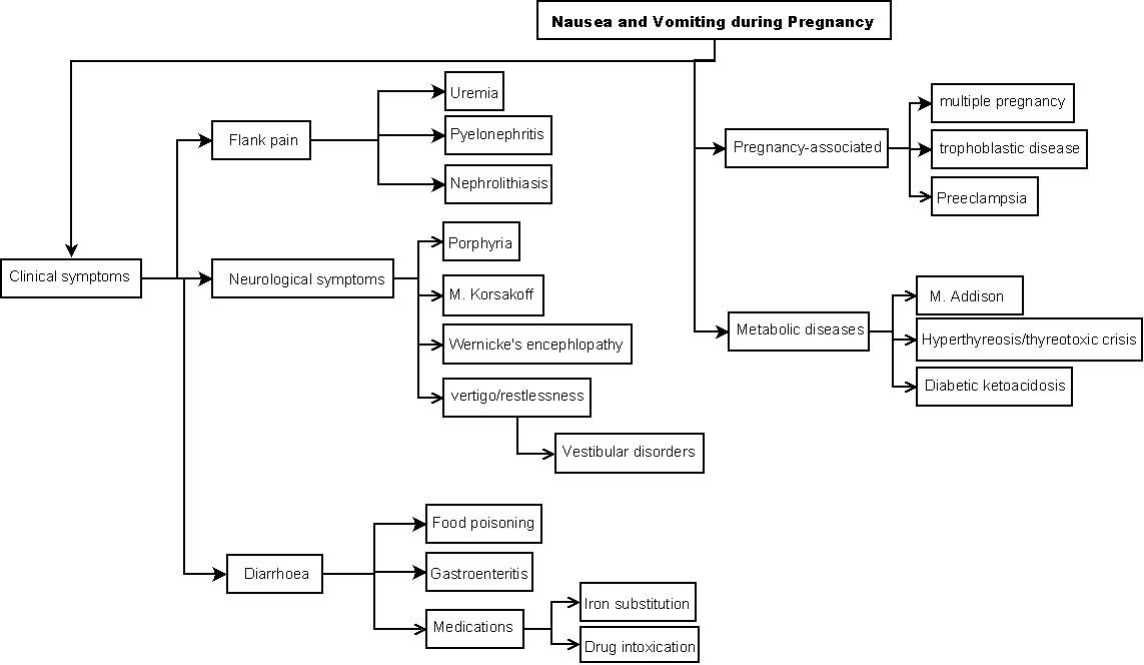
**Differential diagnosis of nausea and vomiting**.

**Figure 2:**
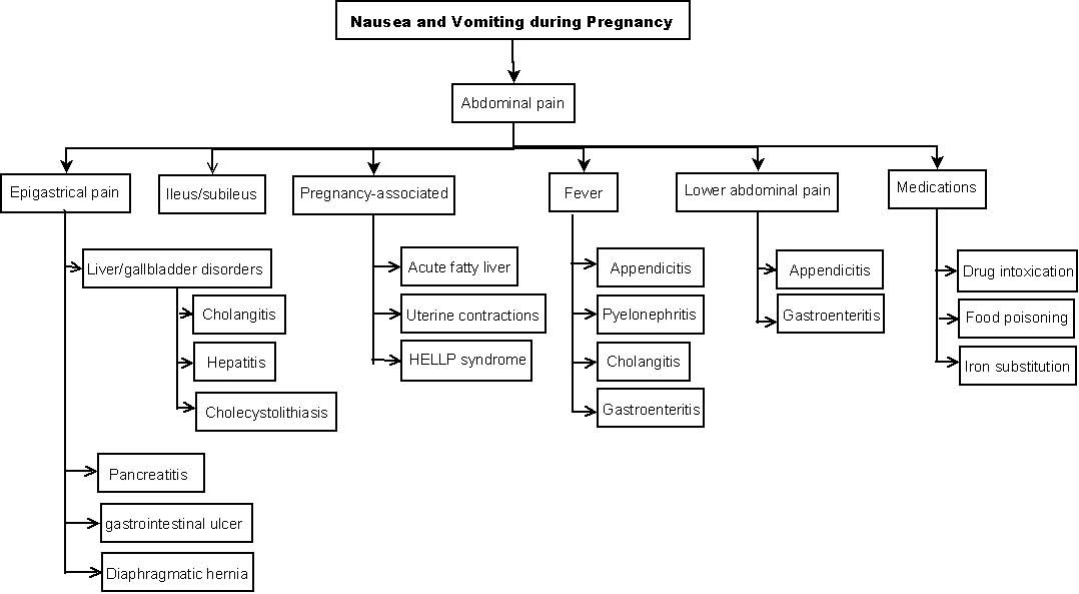
**Differential diagnosis of nausea and vomiting in respect to abdominal symptoms**.

In order to exclude differential diagnoses the following crucial parameters should be investigated: Onset of nausea and vomiting (nearly all of the cases begin before 9 weeks of gestation), attendant symptoms, underlying chronic disorders or, in rare cases, hereditary diseases (see Figures 1 and 2) [4].

A small percentage of pregnant women experience a severe form of nausea and vomiting that is termed HG (synonym: excessive vomiting during pregnancy). This disorder has an estimated incidence of 0.5% - 2% of all live births [5]. A standard definition of HG is the occurrence of more than three episodes of vomiting per day with ketonuria and more than 3 kg or 5% weight loss. However, the diagnosis is usually made clinically following the exclusion of other causes [6,7].

HG can, in individual cases, be life threatening and treatment must be initiated immediately. Clinical findings include dehydration, acidosis due to inadequate nutrition, alkalosis due to loss of hydrochloride and hypokalaemia. There are two degrees of severity: (i) grade 1, nausea and vomiting without metabolic imbalance; and (ii) grade 2, pronounced feelings of sickness with metabolic imbalance [2].

In this article the aetiology, diagnosis, clinical presentation and treatment options will be outlined on the basis of a selective literature review.

## Aetiology

The aetiology of NVP and hyperemesis in pregnancy is unknown, although some biological, physiological and psychological as well as sociocultural factors are thought to be contributory factors [8]. According to another theory, nausea and vomiting during pregnancy might be an evolutionary adaptation that prevents the intake of potentially noxious food. Such harmful substances may be pathogenic microorganisms in meat and toxins in strong-tasting vegetables and beverages. Thus, by avoiding the ingestion of those toxic components, it is supposed that the embryo is protected from miscarriage. Interviews on more than 5400 pregnant women obtained from 20 studies of gestational aversions, as well as enquiries on more than 6200 pregnant women obtained from 21 studies of gestational cravings, show that most women during pregnancy prefer food categories that are less likely to contain toxic substances [9]. However, severe forms of NVP (such as HG) always require medical intervention.

Risk factors for HG include multiple pregnancy, nulliparity, obesity, metabolic disturbances, a history of HG in a previous pregnancy, trophoblastic disorders, psychological disorders (for example, eating disorders such as anorexia nervosa or bulimia) and a history of migration [10-12].

### Human chorionic gonadotrophin (HCG)

HCG is the most likely endocrine factor which accounts for the development of HG. This conclusion is based on observed associations between increased production of HCG (as in molar or in multiple pregnancies) [13-15] and the fact that the incidence of hyperemesis is highest at the time when HCG production reaches its peak during pregnancy (around 9 weeks gestation) [13]. However, there is no evidence to support this hypothesis and some pregnant women do not experience nausea and vomiting despite elevated HCG-levels. In addition, patients suffering from chorionic carcinoma - a disease that is also associated with an increase in HCG - do not usually experience vomiting. These controversial findings may be caused by the varying biological activity of different isoforms of HCG as well as an individual sensitivity for emetogenic stimuli. Additionally, hormone-receptor interactions may modify the effects of HCG leading to hyperemesis in some cases but having no emetic consequences in others [5].

### Helicobacter pylori infection

Chronic infection with helicobacter pylori may also cause HG [16-22]. The histological examination of the gastric mucosa in a total of 30 women (20 HG patients and 10 pregnant volunteers) showed that the bacterium was present in almost 95% of patients with hyperemesis but in only 50% of controls. The differences between the two groups were statistically significant (*P *= 0.009) [16]. In a metaanalysis of 14 case-control studies, which included a total of 1732 patients and controls, an association between the presence of helicobacter pylori and HG was suggested, although not all of the analysed studies reached statistical significance [odds ratio from 0.55 to 109.33, confidence interval (CI) 95%] [6]. Nevertheless, in one study examining both saliva (61.8% detection of helicobacter pylori in patients with HG versus 27.6% in symptom-free pregnant women) and serum (52.9% versus 20.7%) for the bacterium results were significant (*P *< 0.0001, CI 95%) [17], as were cytotoxin-associated gene A and serum findings in a Chinese study [19]. Two observational studies of five patients with HG reported no improvement in symptoms in response to standard drug treatment. However, a marked response was seen following antibiotic treatment (erythromycin) for helicobacter pylori [18,22]. Therefore, although a helicobacter pylori infection might not be the only cause of HG, it should be taken into consideration as a contributing factor in intractable cases of this condition.

### Hormonal factors

Several hormones may cause hyperemesis. These are oestrogen, progesterone, adrenocorticotropic hormone (ACTH), cortisol, growth hormone and prolactin. Evidence for the involvement of serotonin in chemotherapy-induced nausea and vomiting was found some years ago [23,24] implying that this hormone might also play an important role in HG but findings have been inconsistent: In a prospective study on a total of 33 women (13 patients suffering from hyperemesis, 10 healthy pregnant women and 10 nongravid women) serotonin levels were evaluated. The results showed no differences between the three groups [25].

#### Progesterone

Lower [12] and elevated [26] progesterone levels have been reported in patients with hyperemesis. These alterations are caused by pregnancy-associated changes in the mother's immune system: In a prospective study on 44 pregnant women (22 patients with hyperemesis and 22 matching healthy pregnant women) a significant increase in plasma progesterone levels was shown in women with hyperemesis compared to women without the condition (*P *< 0.05). A potentially emetogenic elevation of HCG due to these modifications is proposed [12]. Lower progesterone levels were found in a prospectively controlled study with 62 NVP subjects versus 40 nonaffected pregnant women (*P *< 0.02) [12]. However, several other studies found no association between serum progesterone concentration and hyperemesis [14,27]. Progesterone may cause reduced gastrointestinal motility during pregnancy [28]. Gastric dysrhythmias (tachygastria, bradygastria) may also occur [29], thus contributing to hyperemesis.

#### Oestrogens

Increased levels of oestrogen and oestradiol are known to cause nausea and vomiting in pregnancy [30]. Hence, the presence of a female foetus is associated with severe nausea and vomiting [31,32] usually explained by a raised concentration of oestrogen *in utero *[31]. This is consistent with the observation that oestrogen treatment can cause nausea. It has been suggested that patients with hyperemesis are probably more sensitive to oestrogen effects than asymptomatic pregnant women [12]. Cigarette smokers usually show lower levels of both oestrogens and HCG. Consistently, HG occurs less often in women who smoke [33,34]. However, some studies have reported negative results regarding the association between elevated oestrogen levels and the development of hyperemesis [35].

#### Hyperthyroidism

Thyroid function is physiologically altered during pregnancy, including stimulation by HCG [36]. Hyperthyroidism with normal fT3 and fT4 levels, but decreased levels of thyroid stimulating hormone (TSH), may also be implicated in HG [37,38]. A self-limiting, transient hyperthyroidism of hyperemesis gravidarum (THHG) has been proposed according to the findings of a screening series in 1900 pregnant women who showed markedly increased HCG and fT4 levels [36]. THHG may persist until 18 weeks gestation and does not require treatment. This condition may be partly caused by the high levels of HCG that are often seen in patients with HG since HCG and TSH have a very similar protein structure, thus enabling HCG to act like TRH and hyper-stimulate the thyroid, which recently has been shown conclusively [39,40].

Assignment of a diagnosis of THHG requires the following [2]:

• pathological serology results during hyperemesis

• a negative history of hyperthyroidism before the pregnancy

• the absence of thyroid antibodies

### Psychosomatic approach

Several years ago the theory of underlying psychological alterations as main causative factor for HG was proposed [41]. This was partly thought to be due to a lack of evidence of any straight biological pathophysiological pathway [42], partly due to the finding that many patients seem to need psychological help to cope with the condition.

Not only is their physical identity profoundly changed by the pregnancy but also their daily social life. One former commonly used explication for development of HG was a psychiatric explanation of vomiting as a subconscious wish for an abortion or - in a different approach - a conversion disorder underlying the condition [43].

Although this might be the cause in individual cases, nowadays the generally accepted opinion is that severe psychological afflictions are a consequence of constant vomiting rather than the source of it [8,13]. Such a psychiatric sequel may continue even postpartum [10]. Perhaps as a reaction to the psychological strain of the patients, during the last years psychological treatment approaches seem to increase to a varying extent in different countries [44].

Davis *et al. *found no differences in the incidence of HG in association with marital status, whether or not the pregnancy was desired, or the patient's attitude towards the pregnancy [45]. The most frequently cited study used the psychological Cornell Medical Index to assess 44 pregnant women with, and 49 pregnant women without, HG [35]. A further study used the MMPI to assess patients with HG [46]. Both studies showed that women with hyperemesis had an excessive emotional bond to their mothers and that the main types of personality were hysterical and infantile. Additionally, a systematic questionnaire based on interviews with 23 hospitalized women with HG showed suppressed ambivalent feelings of the expectant mothers towards their pregnancies that were expressed in severe vomiting [47], but this association has not been proven to a sufficient extent [42]. Based on a case control study in more than 11,000 pregnant women an association between pre-pregnancy diagnosed psychiatric conditions (for example, depression, anxiety or drug abuse) and HG was seen (*P *< 0.001) [48]. However, these studies have to be evaluated critically because of a potential methodological weakness [42].

More recent approaches assume that neither a merely physical pathway nor a completely psychological pathomechanism can cause such a complex disease but on the contrary HG is a multifactorial condition. Focusing entirely on psychogenic factors raises the risk that patients are not taken seriously in their suffering and probably not all existing therapeutic options are used. However, neglecting the psychosomatic aspects in the development and course of HG in some patients harbours the risk of treating only the symptoms of the condition without eliminating the cause.

## Medical history and clinical presentation

Clinical symptoms are usually non-specific and it is important to exclude the more unusual causes of nausea and vomiting. These include peptic ulceration, hepatitis, pancreatitis, thyroid disease, gastrointestinal obstruction and adrenocortical insufficiency (see Table 1) [13]. An onset of symptoms after 10 weeks gestation is atypical for NVP and systematic exclusion of the diseases mentioned above is particularly important in such cases.

**Table 1:** Diseases associated with nausea and vomiting during pregnancy (alphabetical order), adapted from reference [2].

Causes	Differential diagnosis
**Gastrointestinal causes**	Appendicitis
	
	Diaphragmatic hernia
	
	Gastroenteritis
	
	Hepatic or cholecystic disorders
	
	Hepatitis
	
	Ileus and subileus
	
	Pancreatitis
	
	Stomach cancer
	
	Stomach ulcer or duodenal ulcer

**Metabolic causes**	Addison's disease
	
	Diabetic ketoacidosis
	
	Hyperthyroidism
	
	Porphyria
	
	Thyrotoxicosis

**Neurological causes**	Korsakoff's psychosis
	
	Migraine
	
	Vestibular disorders
	
	Wernicke's encephalopathy

**Pregnancy associated**	Acute fatty liver
	
	Emesis gravidarum (<5 ×/day)
	
	Hyperemesis gravidarum (>5 ×/day)
	
	Multiple pregancy
	
	Pre-eclampsia
	
	Premature contractions

**Urogenital causes**	Degenerative uterine fibroids
	
	Nephrolithiasis
	
	Pyelonephritis
	
	Uremia

**Other causes**	Drug poisoning
	
	Food poisoning
	
	Iron medication

NVP usually has very few attendant symptoms other than fatigue, exhaustion and indisposition. Pyrexia, gastric pain, headache or neurologic signs point to other causes, although the latter finding may, in rare cases, result from severe and prolonged NVP (for example, Wernicke's encephalopathy or central pontine myelinolysis). In the case of elevated thyroid hormones, it is important to distinguish between THHG and primary thyroid disease. Furthermore, chronic underlying conditions that may have been exacerbated or unmasked by the pregnancy should be excluded [5].

Metabolic ketoacidosis and ketonaemia (acetone-like breath) may also occur with occasional pyrexia and hepatic symptoms (for example, jaundice). In some cases, drowsiness and intellectual slowness are present and this may lead to delirium. Laboratory investigations should include haematocrit, electrolytes, transaminases, bilirubin and thyroid function tests as well as urinary status (presence or absence of ketone bodies, specific weight, pH). In addition, ultrasonography should be performed in order to exclude multiple pregnancy, trophoblastic disorders and neoplasias. If a psychic component of the disorder is suspected, ultrasound scans should not be repeated too often as this may aggravate psychosomatic symptoms [49].

In order to diagnose underlying psychological factors, a psychosomatic dialogue aiming at the formation of a working alliance between patient and physician is essential [50]. This often reveals a stressful and intolerable situation in the patient's social environment that she is unable to escape from or avoid. This psychosocial distress is then converted into a somatic symptom [51]. In the case of persistent vomiting or conspicuous symptoms the psychological differential diagnosis should include deep and defended psychic conflicts [2].

## Treatment strategies

**Figure 3:**
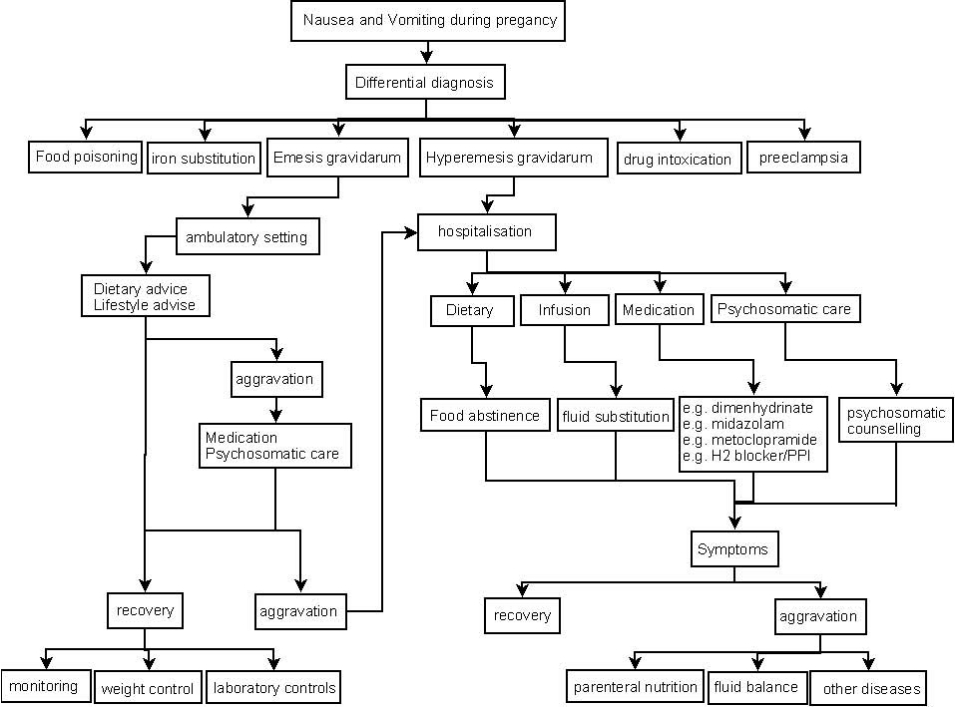
**Multimodal character of treatment strategies in hyperemesis gravidarum, adapted from reference **[2].

Treatment strategies for HG should be based on the severity of symptoms and multimodal in nature (advice, hydration, medication, hospitalization and psychosomatic counselling when necessary; see Figure 3). The severity of the condition can be assessed by numerous questionnaires. Two of the most widely used questionnaires are the Pregnancy-Unique Quantification of Emesis and Nausea (PUQE) scoring index, which assesses nausea and vomiting over 12 h, and the PUQE-24, an extension of the original PUQE, which assesses symptoms over 24 h [52,53]. Another recently developed score is the Hyperemesis Impact of Symptoms Questionnaire (HIS) that brings into focus not only physical but also psychosocial factors in order to assess the impact of HG holistically [54].

### Initial management

For initial management, dietary and lifestyle advice is often sufficient to ameliorate symptoms and improve quality of life. Mild forms of nausea and vomiting can usually be managed by following general nutritional advice such as the intake of small amounts of fluids and food throughout the day rather than eating fewer but larger meals. Foods should be rich on carbohydrates and low in fat and acid [2]. Light snacks, nuts, dairy products, beans and dry and salty biscuits are also frequently recommended. Additionally, electrolyte-replacement drinks and oral nutritional supplements are advisable for ensuring maintenance of electrolyte balance and an adequate intake of calories. Protein-predominant meals have a positive effect because they are eupeptic and are able to reduce nausea more effectively than equicaloric carbohydrate and fat meals or noncaloric meals [55]. If the smell of hot food triggers nausea, cold food should be prepared instead. Lifestyle advice may include avoiding stress and resting in the event of incipient nausea.

Emotional support and, if needed, psychosomatic care administered by a psychologist or a medical doctor with training in psychosomatics can be helpful. Depending on the severity of the symptoms, supportive counselling or crisis intervention might be required [2].

### Medication

If symptoms cannot be managed by dietary and lifestyle changes alone, low dose antiemetics may be administered. All pharmacologic interventions should be based upon known safety, proven efficacy and cost effectiveness [56].

In a metaanalysis of 28 randomized trials of medications for the treatment of HG, antiemetics diminished nausea in early pregnancy and were superior to placebo [2]. Ondansetron is one of the more commonly used and effective drugs and has relatively few side effects [13,57,58].

Other recommended options are the use of metoclopramide to improve gastrointestinal motility and administration of pyridoxine (vitamin B6) [59]. Pyridoxine is given three times daily at a dose of 10 - 25 mg starting with a low dose that may reduce symptoms and has been proven to be more effective than placebo [13,60]. The daily dose can be increased up to 200 mg without side effects [61,62]. However, a more recent placebo-controlled trial demonstrated that a combination of oral pyridoxine and metoclopramide did not improve the vomiting frequency or the nausea score [63].

Antihistamines and anticholinergics such as meclizine, dimenhydrinate and diphenhydramine have also been shown to be superior to placebo and can be used safely for the treatment of nausea and vomiting in pregnancy (see Table 2) [64].

**Table 2:** Antiemetic agents and supposed dosage in hyperemesis gravidarum, adapted from references [13,62,98]

Food and Drug Admnistration category	Medication	Administration	Suggested dosage
**A**	Pyridoxine (vitamin B6)	Oral	20 mg 3 × per day (max. dose: 200 mg per day)
	
	Doxylamine	Oral	25 mg at night and 12.5 mg in the morning accompanied by 10 mg of pyridoxine (maximum dose: 80 mg per day)

**B**	Ondansetron	Oral/intravenously (IV)	4-8 mg 2-3× per day/2-4 mg every 6-8 h or 8 mg every 12 h IV
	
	Metoclopramide	Oral	5-10 mg 3-4 × per day
	
	Meclizine	Oral/rectal	25-100 mg 2-4 × per day/1 × per day
	
	Diphenhydramine	Oral/IV	25-50 mg every 6-8 h
	
	Dimenhydrinate	Oral/IV/rectal	50 mg 3-4× per day/62 mg 2× per day/1-3× per day

**C**	Promethazine	Oral/IV	12.5-25 mg up to 6 × per day
	
	Prochlorperazine	Rectal	25 mg per day or 2× per day
	
	Prednisolone	Oral	40-60 mg per day reducing by half every 3 days

-	Ginger	Oral (biscuits, confectionary, crystals, powder, tablets, capsules, fresh ginger)	Up to 1 g per day in divided doses

However, the side effect profiles vary between the different medications: While nearly all of the above mentioned medicaments may cause dizziness, drowsiness, constipation or dry mouth, more severe adverse effects comprise convulsions, decreased alertness, heartbeat alterations and hallucinations (doxylamine, metoclopramide, diphenhydramine, dimenhydrinate, promethazine). Headache, muscle pain or tremor and fever (prednisolone, procholrperazine, promethazine, dimenhydrinate, doxylamine, metoclopramide) may also occur.

Interestingly, diazepam also has positive effects on HG, probably due to its sedative properties [65]. A prospective randomized clinical trial in 74 patients showed that a combination of antiemetic therapy and diazepam reduced the need for hospitalization and improved patient satisfaction [56]. Frequent use of diazepam, however, might lead to dependency. Another risk factor that must be taken into consideration is the possibility of fetal side effects [2,66]. Antidepressants such as mirtazapine have also been used with some success in ameliorating the symptoms of HG [67].

### Non-pharmacological interventions

Alternative treatments include acupressure, especially on the P6 point (Neiguan) on the inside of the wrist [68,69]. However, despite several trials, there is still minimal experimental evidence that acupuncture is effective in relieving the symptoms of HG., A supplement that may be helpful is ginger. In a trial of 66 women ginger (1 g/day) compared to placebo was shown to be of benefit for those suffering from both nausea and vomiting [70]. Another prospective study of 187 pregnant women with symptoms of NVP showed that ginger had a mild effect in ameliorating nausea and vomiting without negative effects on the fetus [71]. Available data show that ginger has no apparent teratogenic potential and can be used safely up to a daily dose of 1 gram [5,62,72-75].

### Hospitalization

In patients with more severe dehydration or ketonuria, inpatient admission is required (see Figure 3). Sometimes hospitalization alone is sufficient to improve symptoms ('holding function') because it may provide psychological relief. However, the treatment of dehydration is considered to be of paramount importance [13].

The primary therapeutic step is total food withdrawal. Maintaining hydration or, in the case of severe dehydration achieving quick and sufficient rehydration, is the most important intervention. Volume and electrolyte replacement (at least 3 L/day), correction of potential electrolyte imbalance, administration of vitamins and parenteral administration of carbohydrate and amino acid solutions (about 8400 to 10,500 kJ/d) are recommended (see Table 3). Rehydration is most easily and quickly accomplished intravenously and this reduces adverse symptoms very effectively.

**Table 3:** Recommended procedure for substitution of vitamins during total parenteral nutrition (personal communication Ramsauer and Vetter, Berlin, Germany).

Parenteral nutrition via peripheral venous access		
**Main infusion**	**Adjuvants (daily dose)**	**Speed of operation**

500 mL glucose-infusion 5%	• 200 mg vitamin B1 (thiaminchloride), • 200 mg vitamin B6 (pyridoxine), • 200 μg vitamin B12 (cyanocobalamine), • 2000 mg vitamin C (ascorbic acid)	50 mL/h

**Parenteral nutrition via central venous access**		

**Main infusion**	**Adjuvants (daily dose)**	**Speed of operation**

500 mL glucose-infusion 40%	• 200 mg vitamin B1(thiaminchloride), • 200 mg vitamin B6 (pyridoxine), • 200 μg vitamin B12 (cyanocobalamine), • 2000 mg vitamin C (ascorbic acid)	50 mL/h

Although peripheral catheters are considered to be advantageous, a study of 94 patients with HG reported the occurrence of a substantial number of complications in association with peripherally inserted central catheters (PICC lines) compared to treatment with medication only. This suggests that invasive treatment options should be thoroughly investigated before being introduced [76]. Major complications associated with PICC lines are infections and thrombosis or endocarditis. In a retrospective study of 85 pregnant women with central catheters, 25% developed serious complications including infections (12%) [77]. To date, no fetal or maternal medical benefit has been found for PICC lines in comparison to serial peripheral catheters and the use of central catheters is only recommended in exceptional cases [78].

An alternative to parenteral nutrition may be nasogastric feeding tubes because, in cases of intractable nausea and vomiting, they seem to relieve symptoms and provide adequate nutritional support [79]. Another advantage of enteral feeding via nasogastric tubes in comparison to parenteral nutrition is that enteral feeding is more cost effective [80]. In order to determine whether an underlying helicobacter pylori infection is (partially) causative, helicobacter pylori testing may be performed. In the event of a positive result, H2 blockers (for example, cimetidine) or proton pump inhibitors (for example, omeprazol) can be added to infusions [20,81-83].

If hyperemesis is refractory to treatment, corticoids (for example, hydrocortisone) may also be used [84]. Corticosteroids are considered to be safe and have no known adverse effects for the fetus. IV administration may be preferable to oral administration, particularly in refractory cases, as the effect is greater and more rapid [84].

Total parenteral nutrition (TPN) may be useful in highly refractory cases in order to ensure a sufficient calorie intake [85]. However, there is no evidence to support the use of TPN and it should only be used as a last resort when all other treatments have failed, as it can be associated with severe complications such as thrombosis, metabolic disturbances and infection [86].

In patients with persistent symptoms, other disorders should be excluded (see Figures 1 and 2). Treatment should be continued until vomiting ceases or occurs less than three times a day. Subsequent food reintroduction should be carried out gradually [2].

### Psychosomatic therapeutic options

Psychosomatic therapies involve dialogues between the physician and the pregnant woman to evaluate the psychosocial situation in her marital relationship, activate individual resources and provide support regarding acceptance of the pregnancy. On the basis of anamnestic data, other proper therapeutic options such as hypnotherapy, psychotherapy or behavioural therapy may be considered. In approximately 90% of all women hospitalized for HG, symptoms ameliorate without any further therapeutic intervention. This may be due to inpatient nursing support and relief from overstraining, as well as to being removed from the conflict-causing environment. Nevertheless, consideration of psychosocial factors while making the diagnosis may improve long-term treatment outcome [87].

## Pregnancy outcome and prognosis

In most cases, nausea and vomiting during pregnancy is self limiting and is usually resolved by around 20 weeks gestation [13]. Interestingly, it is consistently associated with lower rates of miscarriage, retarded intrauterine growth and preterm delivery and fetal outcomes are, in most cases, excellent [11,88-90].

Other causes of severe distress in these patients include loss of time from work and a reduced quality of life [1]. An investigation of 147 patients evaluated the impact of NVP on family, social and occupational functioning as well as quality of life. It found that 82.8% of patients were limited in their usual daily activities, not only by the presence of constant nausea and frequent vomiting, but also by the psychological burden of not feeling well for several weeks or months [91]. Another stressful aspect of the disorder is the change of the patient's role within her social or occupational environment: Many women feel helpless and incapable and misunderstood by their relatives. Family problems range from neglect of the household to negative alterations in the relationship between the pregnant woman and her spouse. Social functioning is often affected, with a reduction in social commitments, partly due to feelings of embarrassment. Most patients with severe NVP or HG miss at least a few weeks of their paid employment and report that they are less attentive to their work [91].

NVP and HG may cause considerable direct (for example, medication) and indirect (for example, loss of productivity) costs, which can amount to hundreds of dollars. These costs apply not only to the patients, but also to society and the health care system [92]. More serious medical complications include the Mallory-Weiss syndrome (acute increase in oesophageal pressure due to vomiting) and oesophageal rupture (due to severe vomiting), pneumothorax, peripheral neuropathy, coagulopathy, Wernicke's encephalopathy (due to lack of thiamine), pre-eclampsia and fetal growth retardation [11,13,93-95].

The psychosomatic aspects of hyperemesis should also be considered. Interestingly, psychosocial morbidity for the mother often presents in the form of secondary depression, which affects up to 7% of patients with HG [10,96]. Therefore, adequate assessment, observation, and treatment of a possible psychosomatic disorder should also be considered.

## Conclusions

Since the causal factors of HG are multifarious, treatment of this condition should be multimodal, ranging from dietary and lifestyle advice to psychosomatic counselling or psychoanalytic therapy. Administration of antiemetic drugs may be necessary, as well as IV fluid replacement, food administration via nasogastric or parenteral routes in severe cases. Since this condition is accompanied by a significant reduction in quality of life for the patient and high costs to the healthcare system, general practitioners and obstetricians should ensure that they are well informed about this condition [97] so that they are able to provide advice, counselling and effective medication to pregnant women and thus prevent the exacerbation of symptoms.

## Abbreviations

ACTH: adrenocorticotropic hormone; HCG: human chorionic gonadotrophin; HG: hyperemesis gravidarum; IV: intravenous; MMPI: Minnesota Multiphasic Personality Inventory; NVP: nausea and vomiting during pregnancy; PICC: peripherally inserted central catheter; PUQE: Pregnancy-Unique Quantification of Emesis and Nausea; THHG: hyperthyroidism of HG; TPN: total parenteral nutrition; TSH: thyroid stimulating hormone.

## Competing interests

The authors declare that they have no competing interests.

## Authors' contributions

JJ and IM designed, conducted the literature review and drafted most of the manuscript. RK wrote the psychosomatic part of this article. All authors read and approved the final manuscript.

## Pre-publication history

The pre-publication history for this paper can be accessed here:

http://www.biomedcentral.com/1741-7015/8/46/prepub

## References

[B1] Gadsby R Barnie-Adshead AM Jagger C A prospective study of nausea and vomiting during pregnancy Br J Gen Pract 1993 43 245 248 8373648 PMC1372422

[B2] Mylonas I Gingelmaier A Kainer F Nausea and vomiting in pregnancy Dtsch Arztebl 2007 104 A1821 1826

[B3] Klebanoff MA Koslowe PA Kaslow R Rhoads GG Epidemiology of vomiting in early pregnancy Obstet Gynecol 1985 66 5 612 616 3903578

[B4] Rodien P Bremont C Sanson ML Parma J Van Sande J Costagliola S Luton JP Vassart G Duprez L Familial gestational hyperthyroidism caused by a mutant thyrotropin receptor hypersensitive to human chorionic gonadotropin N Engl J Med 1998 339 1823 1826 10.1056/NEJM199812173392505 9854118

[B5] ACOG (American College of Obstetrics and Gynecology) Practice bulletin: nausea and vomiting of pregnancy Obstet Gynecol 2004 103 4 803 814 15051578

[B6] Golberg D Szilagyi A Graves L Hyperemesis gravidarum and *Helicobacter pylori *infection: a systematic review Obstet Gynecol 2007 110 695 703 17766620 10.1097/01.AOG.0000278571.93861.26

[B7] Gadsby R Barnie-Adshead AM Jaggerc C Pregnancy nausea related to women's obstetric and personal histories Gynecol Obstet Invest 1997 43 108 111 10.1159/000291833 9067717

[B8] Simpson SW Goodwin TM Robins SB Rizzo AA Howes RA Buckwalter DK Buckwalter JG Psychological factors and hyperemesis gravidarum J Womens Health Gend Based Med 2001 10 471 477 10.1089/152460901300233948 11445046

[B9] Sherman PW Flaxman SM Nausea and vomiting of pregnancy in an evolutionary perspective Am J Obstet Gynecol 2002 186 S190 7 10.1067/mob.2002.122593 12011885

[B10] Poursharif B Korst LM Fejzo MS MacGibbon KW Romero R Goodwin TM The psychosocial burden of hyperemesis gravidarum J Perinatol 2008 28 176 181 10.1038/sj.jp.7211906 18059463

[B11] Broussard CN Richter JE Nausea and vomiting of pregnancy Gastroenterol Clin North Am 1998 27 123 151 10.1016/S0889-8553(05)70350-2 9546087

[B12] Jarnfelt-Samsioe A Nausea and vomiting in pregnancy: a review Obstet Gynecol Surv 1987 42 422 427 10.1097/00006254-198707000-00003 3614796

[B13] Sheehan P Hyperemesis gravidarum--assessment and management Aust Fam Physician 2007 36 698 701 17885701

[B14] Masson GM Anthony F Chau E Serum chorionic gonadotrophin (hCG), schwangerschaftsprotein 1 (SP1), progesterone and oestradiol levels in patients with nausea and vomiting in early pregnancy Br J Obstet Gynaecol 1985 92 211 215 3872132 10.1111/j.1471-0528.1985.tb01084.x

[B15] Goodwin TM Hershman JM Cole L Increased concentration of the free beta-subunit of human chorionic gonadotropin in hyperemesis gravidarum Acta Obstet Gynecol Scand 1994 73 770 772 10.3109/00016349409072502 7817726

[B16] Bagis T Gumurdulu Y Kayaselcuk F Yilmaz ES Killicadag E Tarim E Endoscopy in hyperemesis gravidarum and *Helicobacter pylori *infection Int J Gynaecol Obstet 2002 79 105 109 10.1016/S0020-7292(02)00230-8 12427393

[B17] Hayakawa S Nakajima N Karasaki-Suzuki M Yoshinaga H Arakawa Y Satoh K Yamamoto T Frequent presence of *Helicobacter pylori *genome in the saliva of patients with hyperemesis gravidarum Am J Perinatol 2000 17 243 247 10.1055/s-2000-10005 11110341

[B18] Jacoby EB Porter KB *Helicobacter pylori *infection and persistent hyperemesis gravidarum Am J Perinatol 1999 16 85 88 10.1055/s-2007-993841 10355915

[B19] Xia LB Yang J Li AB Tang SH Xie QZ Cheng D Relationship between hyperemesis gravidarum and *Helicobacter pylori *seropositivity Chin Med J (Engl) 2004 117 301 302 14975221

[B20] Sandven I Abdelnoor M Nesheim BI Melby KK *Helicobacter pylori *infection and hyperemesis gravidarum: a systematic review and meta-analysis of case-control studies Acta Obstet Gynecol Scand 2009 88 1190 1200 10.3109/00016340903284927 19900137

[B21] Kazerooni T Taallom M Ghaderi AA *Helicobacter pylori *seropositivity in patients with hyperemesis gravidarum Int J Gynaecol Obstet 2002 79 217 220 10.1016/S0020-7292(02)00298-9 12445985

[B22] El Younis CM Abulafia O Sherer DM Rapid marked response of severe hyperemesis gravidarum to oral erythromycin Am J Perinatol 1998 15 533 534 10.1055/s-2007-994055 9890250

[B23] Cubeddu LX Hoffmann IS Fuenmayor NT Finn AL Efficacy of ondansetron (GR 38032F) and the role of serotonin in cisplatin-induced nausea and vomiting N Engl J Med 1990 322 810 816 1689807 10.1056/NEJM199003223221204

[B24] Wilder-Smith OH Borgeat A Chappuis P Fathi M Forni M Urinary serotonin metabolite excretion during cisplatin chemotherapy Cancer 1993 72 2239 2241 10.1002/1097-0142(19931001)72:7<2239::AID-CNCR2820720729>3.0.CO;2-5 7690681

[B25] Borgeat A Fathi M Valiton A Hyperemesis gravidarum: is serotonin implicated? Am J Obstet Gynecol 1997 176 476 477 10.1016/S0002-9378(97)70518-0 9065201

[B26] Yoneyama Y Suzuki S Sawa R Yoneyama K Doi D Otsubo Y Araki T The T-helper 1/T-helper 2 balance in peripheral blood of women with hyperemesis gravidarum Am J Obstet Gynecol 2002 187 1631 1635 10.1067/mob.2002.127373 12501075

[B27] Lagiou P Tamimi R Mucci LA Trichopoulos D Adami HO Hsieh CC Nausea and vomiting in pregnancy in relation to prolactin, estrogens, and progesterone: a prospective study Obstet Gynecol 2003 101 639 644 10.1016/S0029-7844(02)02730-8 12681864

[B28] Koch KL Frissora CL Nausea and vomiting during pregnancy Gastroenterol Clin North Am 2003 32 201 234 vi 10.1016/S0889-8553(02)00070-5 12635417

[B29] Walsh JW Hasler WL Nugent CE Owyang C Progesterone and estrogen are potential mediators of gastric slow-wave dysrhythmias in nausea of pregnancy Am J Physiol 1996 270 G506 514 8638718 10.1152/ajpgi.1996.270.3.G506

[B30] Depue RH Bernstein L Ross RK Judd HL Henderson BE Hyperemesis gravidarum in relation to estradiol levels, pregnancy outcome, and other maternal factors: a seroepidemiologic study Am J Obstet Gynecol 1987 156 1137 1141 3578425 10.1016/0002-9378(87)90126-8

[B31] Schiff MA Reed SD Daling JR The sex ratio of pregnancies complicated by hospitalisation for hyperemesis gravidarum BJOG 2004 111 27 30 10.1046/j.1471-0528.2003.00005.x 14687048

[B32] James WH The associated offspring sex ratios and cause(s) of hyperemesis gravidarum Acta Obstet Gynecol Scand 2001 80 378 379 11264620 10.1034/j.1600-0412.2001.080004378.x

[B33] Bernstein L Pike MC Lobo RA Depue RH Ross RK Henderson BE Cigarette smoking in pregnancy results in marked decrease in maternal hCG and oestradiol levels Br J Obstet Gynaecol 1989 96 92 96 2923845 10.1111/j.1471-0528.1989.tb01582.x

[B34] Baron JA La Vecchia C Levi F The antiestrogenic effect of cigarette smoking in women Am J Obstet Gynecol 1990 162 502 514 2178432 10.1016/0002-9378(90)90420-c

[B35] Fairweather DV Nausea and vomiting during pregnancy Obstet Gynecol Annu 1978 7 91 105 662219

[B36] Glinoer D The regulation of thyroid function in pregnancy: pathways of endocrine adaptation from physiology to pathology Endocr Rev 1997 18 404 33 10.1210/er.18.3.404 9183570

[B37] Chan NN Thyroid function in hyperemesis gravidarum Lancet 1999 353 2243 10.1016/S0140-6736(05)76292-9 10393011

[B38] Blankenstein TJ Kainer F Friese K Mylonas I Extended hyperemesis gravidarum in a patient after total thyroidectomy Arch Gynecol Obstet 2009 280 1029 1031 10.1007/s00404-009-1026-z 19322577

[B39] Rodien P Jordan N Lefèvre A Royer J Vasseur C Savagner F Bourdelot A Rohmer V Abnormal stimulation of the thyrotrophin receptor during gestation Hum Reprod Update 2004 10 2 95 105 10.1093/humupd/dmh008 15073140

[B40] Yoshimura M Hershman JM Thyrotropic action of human chorionic gonadotropin Thyroid 1995 5 5 425 34 10.1089/thy.1995.5.425 8563483

[B41] Buckwalter JG Simpson SW Psychological factors in the etiology and treatment of severe nausea and vomiting in pregnancy Am J Obstet Gynecol 2002 186 S210 214 10.1067/mob.2002.122600 12011888

[B42] Munch S Chicken or the egg? The biological-psychological controversy surrounding hyperemesis gravidarum Soc Sci Med 2002 55 1267 1278 10.1016/S0277-9536(01)00239-8 12365536

[B43] El-Mallakh RS Liebowitz NR Hale MS Hyperemesis gravidarum as conversion disorder J Nerv Ment Dis 1990 178 655 659 10.1097/00005053-199010000-00007 2230751

[B44] Goodwin TM Poursharif B Korst LM MacGibbon KW Romero R Fejzo MS Secular trends in the treatment of hyperemesis gravidarum Am J Perinatol 2008 25 141 147 10.1055/s-2008-1040344 18260047

[B45] Davis M Nausea and vomiting of pregnancy: an evidence-based review J Perinat Neonatal Nurs 2004 18 312 328 15646303 10.1097/00005237-200410000-00002

[B46] Fairweather DV Nausea and vomiting in pregnancy Am J Obstet Gynecol 1968 102 135 175 4877794 10.1016/0002-9378(68)90445-6

[B47] Karpel L de Gmeline C Psychological approach to hyperemis gravidarum J Gynecol Obstet Biol Reprod (Paris) 2004 33 623 631 15550881 10.1016/s0368-2315(04)96603-3

[B48] Seng JS Schrot JA van De Ven C Liberzon I Service use data analysis of pre-pregnancy psychiatric and somatic diagnoses in women with hyperemesis gravidarum J Psychosom Obstet Gynaecol 2007 28 209 217 10.1080/01674820701262044 17852658

[B49] Kirk E Papageorghiou AT Condous G Bottomley C Bourne T Hyperemesis gravidarum: is an ultrasound scan necessary? Hum Reprod 2006 21 2440 2442 10.1093/humrep/del166 16720621

[B50] Adler RH Uexküll T Anamnese und körperliche Untersuchung Psychosomatische Medizin: Modelle ärztlichen Denkens und Handelns 2008 Munich/Jena: Elsevier 397 412

[B51] Wenderlin JM Stauber M Hyperemesis as a psychosomatisches problem Psychosomatische Geburtshilfe und Gynäkologie 1999 Berlin: Springer 117 124

[B52] Lacasse A Rey E Ferreira E Morin C Berard A Validity of a modified Pregnancy-Unique Quantification of Emesis and Nausea (PUQE) scoring index to assess severity of nausea and vomiting of pregnancy Am J Obstet Gynecol 2008 198 e71 77 10.1016/j.ajog.2007.05.051 18166311

[B53] Ebrahimi N Maltepe C Bournissen FG Koren G Nausea and vomiting of pregnancy: using the 24-hour Pregnancy-Unique Quantification of Emesis (PUQE-24) scale J Obstet Gynaecol Can 2009 31 9 803 807 19941704 10.1016/S1701-2163(16)34298-0

[B54] Power Z Campbell M Kilcoyne P Kitchener H Waterman H The Hyperemesis Impact of Symptoms Questionnaire: development and validation of a clinical tool Int J Nurs Stud 2010 47 1 67 77 10.1016/j.ijnurstu.2009.06.012 19646694

[B55] Jednak M Shadigian EM Kim MS Woods ML Hooper FG Owyang C Hasler WL Protein meals reduce nausea and gastric slow wave dysrhythmic activity in first trimester pregnancy Am J Physiol 1999 277 4 Pt1 G855 861 10516152 10.1152/ajpgi.1999.277.4.G855

[B56] Reichmann JP Kirkbride MS Nausea and vomiting of pregnancy: cost effective pharmacologic treatments Manag Care 2008 17 41 45 19127765

[B57] World MJ Ondansetron and hyperemesis gravidarum Lancet 1993 341 8838 185 10.1016/0140-6736(93)90054-K 8093793

[B58] Einarson A Maltepe C Navioz Y Kennedy D Tan MP Koren G The safety of ondansetron for nausea and vomiting of pregnancy: a prospective comparative study BJOG 2004 111 940 943 10.1111/j.1471-0528.2004.00236.x 15327608

[B59] Matok I Gorodischer R Koren G Sheiner E Wiznitzer A Levy A The safety of metoclopramide use in the first trimester of pregnancy N Engl J Med 2009 360 24 2528 35 10.1056/NEJMoa0807154 19516033

[B60] Sahakian V Rouse D Sipes S Rose N Niebyl J Vitamin B6 is effective therapy for nausea and vomiting of pregnancy: a randomized, double-blind placebo-controlled study Obstet Gynecol 1991 78 33 36 2047064

[B61] Shrim A Boskovic R Maltepe C Navios Y Garcia-Bournissen F Koren G Pregnancy outcome following use of large doses of vitamin B6 in the first trimester J Obstet Gynaecol 2006 26 8 749 751 10.1080/01443610600955826 17130022

[B62] Einarson A Maltepe C Boskovic R Koren G Treatment of nausea and vomiting in pregnancy: an updated algorithm Can Fam Physician 2007 53 12 2109 2111 18077743 PMC2231543

[B63] Tan PC Yow CM Omar SZ A placebo-controlled trial of oral pyridoxine in hyperemesis gravidarum Gynecol Obstet Invest 2009 67 151 157 10.1159/000181182 19077388

[B64] Leathem AM Safety and efficacy of antiemetics used to treat nausea and vomiting in pregnancy Clin Pharm 1986 5 660 668 2874910

[B65] Ditto A Morgante G la Marca A De Leo V Evaluation of treatment of hyperemesis gravidarum using parenteral fluid with or without diazepam. A randomized study Gynecol Obstet Invest 1999 48 232 236 10.1159/000010189 10592423

[B66] Tasci Y Demir B Dilbaz S Haberal A Use of diazepam for hyperemesis gravidarum J Matern Fetal Neonatal Med 2009 22 353 356 10.1080/14767050802464528 19089776

[B67] Schwarzer V Heep A Gembruch U Rohde A Treatment resistant hyperemesis gravidarum in a patient with type 1 diabetes mellitus: neonatal withdrawal symptoms after successful antiemetic therapy with mirtazapine Arch Gynecol Obstet 2008 277 1 67 69 10.1007/s00404-007-0406-5 17628816

[B68] de Aloysio D Penacchioni P Morning sickness control in early pregnancy by Neiguan point acupressure Obstet Gynecol 1992 80 852 854 1407927

[B69] Shin HS Song YA Seo S Effect of Nei-Guan point (P6) acupressure on ketonuria levels, nausea and vomiting in women with hyperemesis gravidarum J Adv Nurs 2007 59 510 519 10.1111/j.1365-2648.2007.04342.x 17645494

[B70] Vutyavanich T Kraisarin T Ruangsri R Ginger for nausea and vomiting in pregnancy: randomized, double-masked, placebo-controlled trial Obstet Gynecol 2001 97 577 582 10.1016/S0029-7844(00)01228-X 11275030

[B71] Portnoi G Chng LA Karimi-Tabesh L Koren G Tan MP Einarson A Prospective comparative study of the safety and effectiveness of ginger for the treatment of nausea and vomiting in pregnancy Am J Obstet Gynecol 2003 189 5 1374 1377 10.1067/S0002-9378(03)00649-5 14634571

[B72] Nordeng H Havnen GC Use of herbal drugs in pregnancy: a survey among 400 norwegian women Pharmacoepidemiol Drug Saf 2004 13 6 371 380 10.1002/pds.945 15170766

[B73] Borrelli F Capasso R Aviello G Pittler MH Izzo AA Effectiveness and safety of ginger in the treatment of pregnancy-induced nausea and vomiting Obstet Gynecol 2005 105 4 849 856 15802416 10.1097/01.AOG.0000154890.47642.23

[B74] Mills E Duguoa JJ Perri D Koren G Herbal medicines in pregnancy and lactation: an evidence-based approach 2006 New York: Taylor and Francis

[B75] Ozgoli G Goli M Simbar M Effects of ginger capsules on pregnancy, nausea, and vomiting J Altern Complement Med 2009 15 3 243 246 10.1089/acm.2008.0406 19250006

[B76] Holmgren C Aagaard-Tillery KM Silver RM Porter TF Varner M Hyperemesis in pregnancy: an evaluation of treatment strategies with maternal and neonatal outcomes Am J Obstet Gynecol 2008 198 56 e51-54. 10.1016/j.ajog.2007.06.004 18166306

[B77] Nuthalapaty FS Beck MM Mabie WC Complications of central venous catheters during pregnancy and postpartum: a case series Am J Obstet Gyneco 2009 201 3 311.e1-5. 10.1016/j.ajog.2009.06.020 19733284

[B78] Ogura JM Francois KE Perlow JH Elliott JP Complications associated with peripherally inserted central catheter use during pregnancy Am J Obstet Gynecol 2003 188 5 1223 1225 10.1067/mob.2003.332 12748485

[B79] Hsu JJ Clark-Glena R Nelson DK Kim CH Nasogastric enteral feeding in the management of hyperemesis gravidarum Obstet Gynecol 1996 88 343 6 10.1016/0029-7844(96)00174-3 8752236

[B80] van de Ven CJ Nasogastric enteral feeding in hyperemesis gravidarum Lancet 1997 349 9050 445 446 10.1016/S0140-6736(05)61177-4 9040568

[B81] Gill SK O'Brien L Koren G The safety of histamine 2 (H2) blockers in pregnancy: a meta-analysis Dig Dis Sci 2009 54 9 1835 1838 10.1007/s10620-008-0587-1 19051023

[B82] Gill SK The safety of proton pump inhibitors (PPIs) in pregnancy: a meta-analysis Am J Gastroenterol 2009 104 6 1541 1545 10.1038/ajg.2009.122 19491869

[B83] Gill SK The effect of acid-reducing pharmacotherapy on the severity of nausea and vomiting of pregnancy Obstet Gynecol Int 2009 1 4 Article ID 585269 10.1155/2009/585269 PMC2778456 19960057

[B84] Nelson-Piercy C Fayers P de Swiet M Randomised, double-blind, placebo-controlled trial of corticosteroids for the treatment of hyperemesis gravidarum BJOG 2001 108 9 15 10.1016/S0306-5456(00)00017-6 11213010

[B85] Levine MG Esser D Total parenteral nutrition for the treatment of severe hyperemesis gravidarum: maternal nutritional effects and fetal outcome Obstet Gynecol 1988 72 1 102 107 3132667

[B86] Ismail SK Kenny L Review on hyperemesis gravidarum Best Pract Res Clin Gastroenterol 2007 21 5 755 769 10.1016/j.bpg.2007.05.008 17889806

[B87] Leeners B Sauer I Rath W Nausea and vomiting in early pregnancy/hyperemesis gravidarum. Current status of psychosomatic factors Z Geburtshilfe Neonatol 2000 204 128 134 10.1055/s-2000-10209 11008334

[B88] Weigel RM Weigel MM Nausea and vomiting of early pregnancy and pregnancy outcome. A meta-analytical review Br J Obstet Gynaecol 1989 96 1312 1318 2611170 10.1111/j.1471-0528.1989.tb03229.x

[B89] Tsang IS Katz VL Wells SD Maternal and fetal outcomes in hyperemesis gravidarum Int J Gynaecol Obstet 1996 55 231 235 10.1016/S0020-7292(96)02778-6 9003948

[B90] Brandes JM First-trimester nausea and vomiting as related to outcome of pregnancy Obstet Gynecol 1967 30 427 431 6037702

[B91] O'Brien B Naber S Nausea and vomiting during pregnancy: effects on the quality of women's lives Birth 1992 19 138 143 10.1111/j.1523-536X.1992.tb00671.x 1388440

[B92] Piwko C Ungar WJ Einarson TR Wolpin J Koren G The weekly cost of nausea and vomiting of pregnancy for women calling the Toronto Motherisk Program Curr Med Res Opin 2007 23 4 833 840 10.1185/030079907X178739 17407640

[B93] Netravathi M Sinha S Taly AB Bindu PS Bharath RD Hyperemesis gravidarum induced Wernicke's encephalopathy: serial clinical, electrophysiological and MR imaging observations J Neurol Sci 2009 284 214 216 10.1016/j.jns.2009.05.004 19477464

[B94] Zhang J Cai WW Severe vomiting during pregnancy: antenatal correlates and fetal outcomes Epidemiology 1991 2 454 457 10.1097/00001648-199111000-00013 1790200

[B95] Francini-Pesenti F Brocadello F Manara R Santelli L Laroni A Caregaro L Wernicke's syndrome during parenteral feeding: not an unusual complication Nutrition 2009 25 142 146 10.1016/j.nut.2008.08.003 18929463

[B96] Goodwin TM Nausea and vomiting of pregnancy: an obstetric syndrome Am J Obstet Gynecol 2002 186 Suppl 5 184 189 12011884 10.1067/mob.2002.122592

[B97] Power ML Milligan LA Schulkin J Managing nausea and vomiting of pregnancy: a survey of obstetrician-gynecologists J Reprod Med 2007 52 922 928 17977167

[B98] Atanackovic G Navioz Y Moretti ME Koren G The safety of higher than standard dose of doxylamine-pyridoxine (Diclectin) for nausea and vomiting of pregnancy J ClinPharmacol 2001 41 8 842 845 10.1177/00912700122010735 11504271

